# Supplementary material for: Dendrimer-Conjugated nSMase2 Inhibitor Reduces Tau Propagation in Mice
Source: Pharmaceutics. 2022 Sep 28;14(10):2066. doi: 10.3390/pharmaceutics14102066 (PMC9609094; doi:10.3390/pharmaceutics14102066)
Supplement: Supplementary file 1 [file pharmaceutics-14-02066-s001.zip › pharmaceutics-1904777-supplementary.pdf]

Supplementary Information

Supplementary Methods

Synthesis of compound 2: DMAP (161mg, 1.32mmoles) and EDC (303mg, 1.58mmoles) were added to a stirring solution of DPTIP (compound **1**, 500mg, 1.32mmoles) in anhydrous dichloromethane (DCM, 20mL) for 10 minutes. This was followed by the addition of azido-PEG-4-acid (461mg, 1.58mmoles). The solution was stirred at room temperature overnight. Upon completion, the reaction mixture was diluted with DCM (20mL) and washed with water followed by brine. The organic layer was dried over anhydrous sodium sulfate, decanted, and evaporated under vacuum. The crude product was purified via silica-gel based column chromatography using DCM in methanol (MeOH). The pure fractions were obtained in 5% MeOH in DCM, and evaporated to afford compound **2** as viscous liquid. Yield: 88%.

^{1}H NMR (500 MHz, DMSO) δ 7.66 – 7.58 (m, 2H), 7.57 – 7.50 (m, 2H), 7.50 – 7.44 (m, 1H), 7.43 (s, 1H), 7.41-7.31 (m, 2H), 7.30 – 7.17 (m, 1H), 7.02-6.94 (m, 1H), 3.86 (s, 6H), 3.76 (t, 2H, J = 6.2 Hz), 3.61 (dd, J = 10.0, 4.9 Hz, 2H), 3.59 – 3.48 (m, 12H), 3.40 (m, 2H), 2.83 (t, J = 6.2 Hz, 2H). (**Supplementary Figure S1**)

HRMS: m/z: calculated: 651.74, found: 652.24 $[M+1]^+$ (**Supplementary Figure S2**)

Synthesis of compound 4: 5-hexynoic acid (157mg, 1.4mmoles) was added to a stirring solution of PAMAM-G_4-OH (**3**, 2.00g, 0.140 mmoles) in anhydrous DMF, followed by the addition of DMAP (170mg, 1.4mmoles) and EDC (401mg, 2.1mmoles), and stirred for 48 hours at room temperature. The reaction mixture was then diluted with DMF (200mL), and dialyzed against DMF for 4 hours followed by the dialysis against water for 12 hours. During dialysis, the solvents were changed frequently. The final aqueous solution was freeze-dried to afford D-Hexyne (compound **4**) as white hygroscopic solid. Yield: 87%.

^{1}H NMR (500 MHz, D_2O) δ 8.25-7.65 (m, D-internal amide *H*), 4.72 (bs, D-O*H*), 4.01 (t, ester –CH_2), 3.49 – 3.21 (m, D-–CH_2), 3.11 (m, D- and linker–CH_2), 2.79 – 2.58 (m, D- and linker–CH_2), 2.49-2.30 (m, D–CH_2), 2.31-2.05 (m, D–CH_2), 1.69 (t, linker–CH_2). (**Supplementary Figure S3**)

HPLC: Retention time: 8.5 minutes, Purity >99% (**Supplementary Figure S4**)

Supplementary Figures

Characterization of intermediates and dendrimer conjugates:

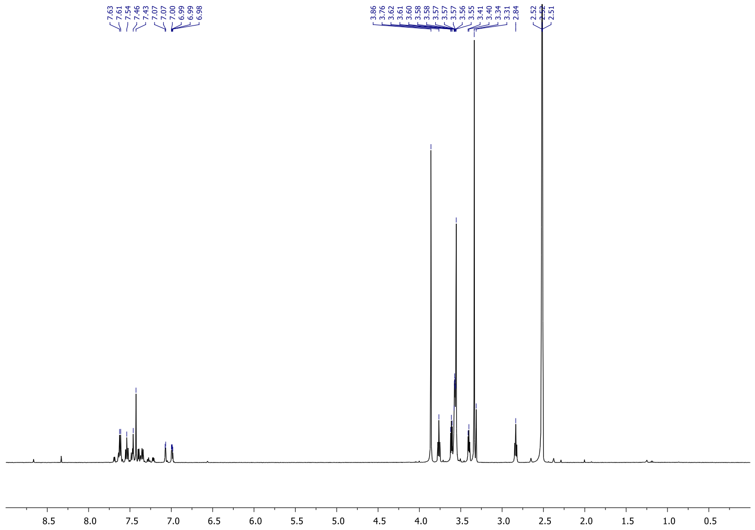

Figure S1. ^{1}H NMR of compound **2** (DPTIP-azide) (DMSO-*d*6; 500MHz).

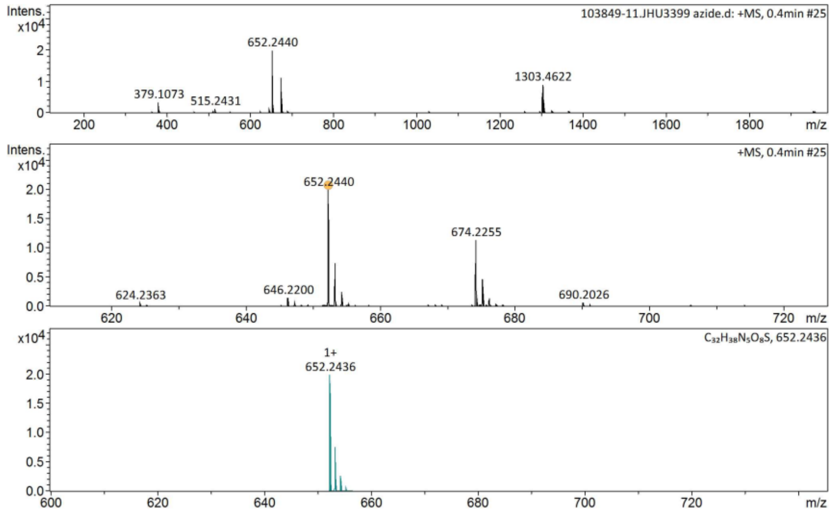

Figure S2. ESI-MS spectrum of compound **2 (**DPTIP-azide) (+ve mode)

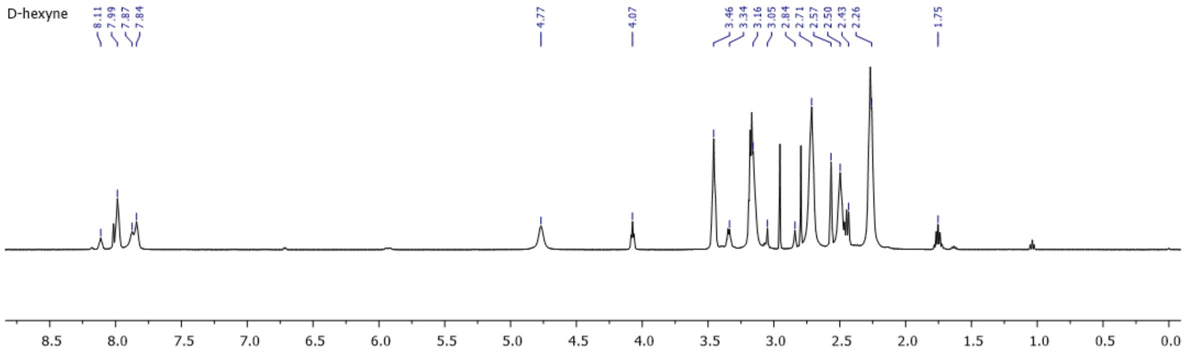

Figure S3. ^{1}H NMR of compound **4** (D_4-Hexyne) (DMSO-*d*6; 500MHz).

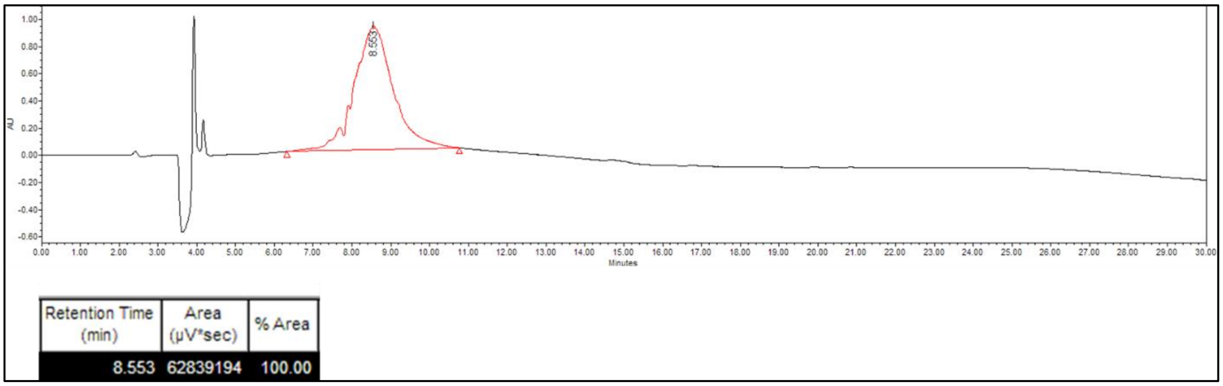

Retention Time (min)	Area (µV*sec)	% Area
8.553	62839194	100.00

Figure S4. HPLC chromatogram of compound **4** (D_4-Hexyne) at 210nm.

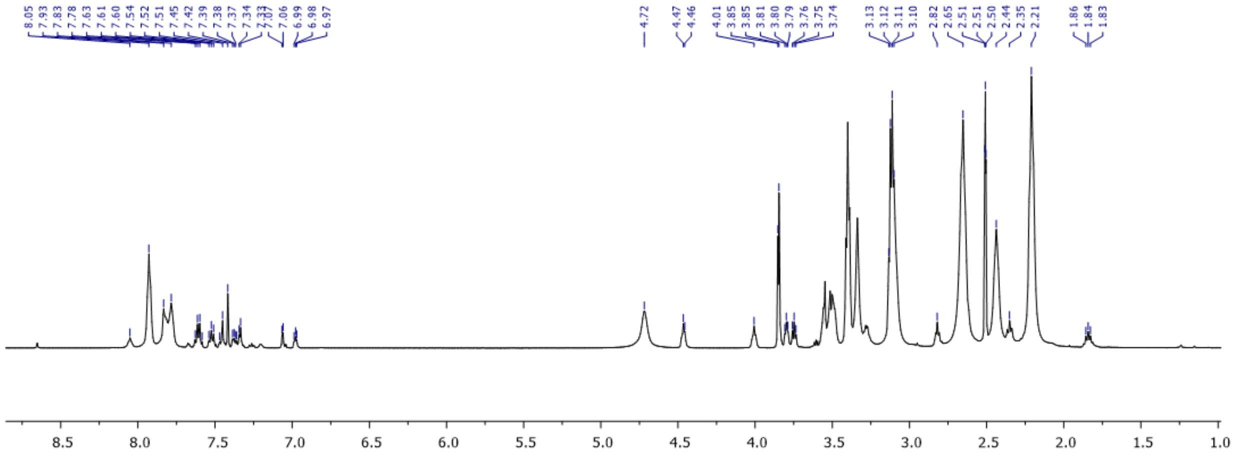

Figure S5. ^{1}H NMR of compound **5** (D-DPTIP) (DMSO-*d*6; 500MHz).

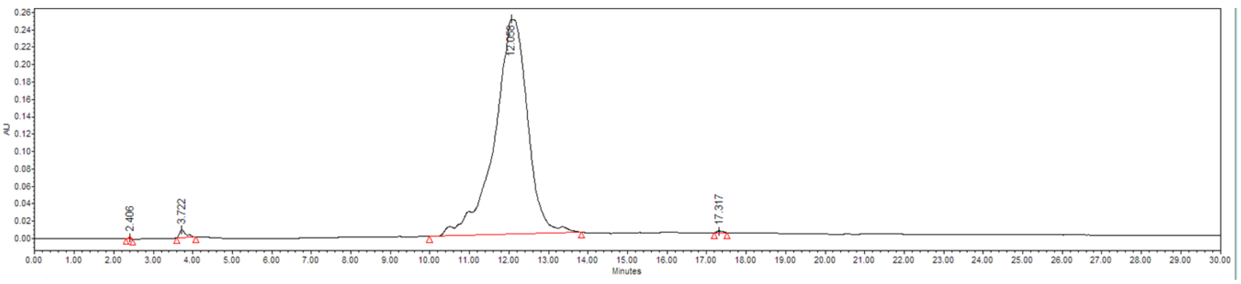

Retention Time (min)	Area (µV*sec)	% Area
2.406	9424	0.06
3.722	97011	0.67
12.058	14369842	99.10
17.317	23882	0.16

Figure S6. HPLC chromatogram of compound **5** (D-DPTIP) at 280nm.

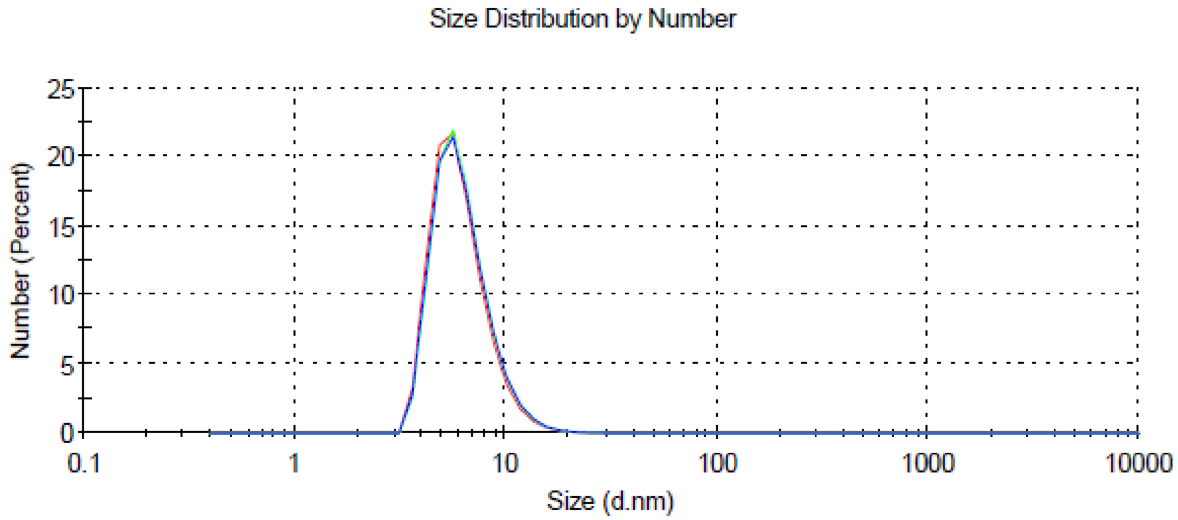

Figure S7. Size distribution of compound **5** (D-DPTIP) via dynamic light scattering (n=3): 6.3±0.1 nm.

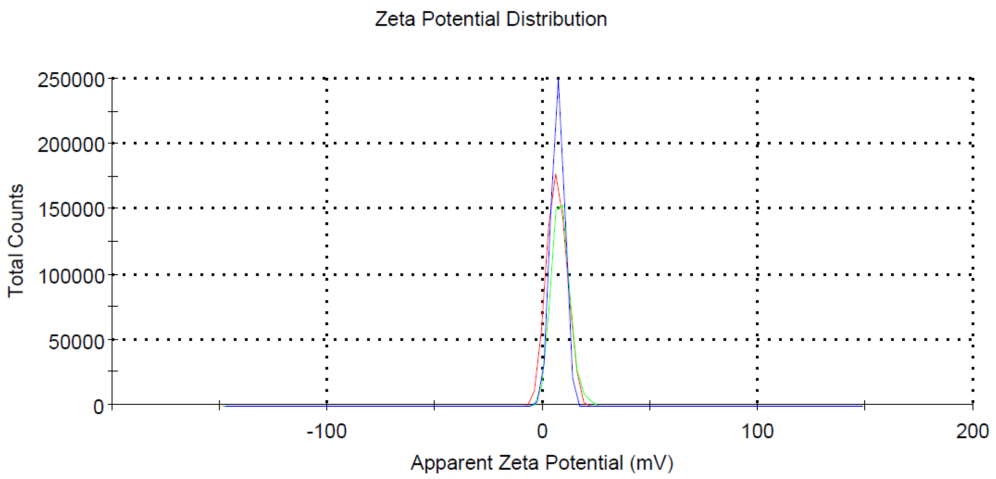

Figure S8. Zeta potential distribution of compound **5 (**D-DPTIP) via dynamic light scattering (n=3): +7.2±0.9 mV.

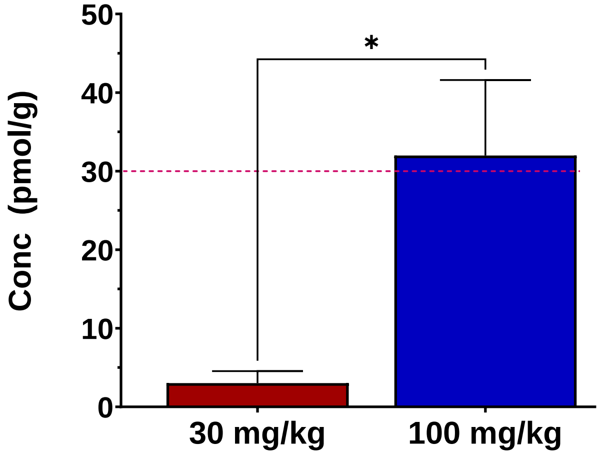

Figure S9. Brain levels of DPTIP in mice dosed with oral 30mg/kg and 100mg/kg D-DPTIP (DPTIP equivalent) at 24h post administration. Bars represent mean ± SEM. Red dotted line indicates DPTIP IC_{50} (30nM). * < 0.05.

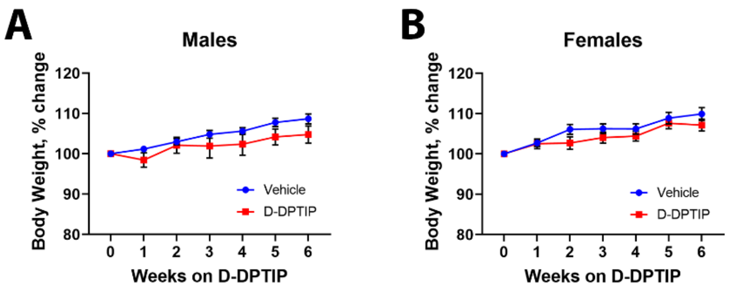

C

Parameter	Males			Females		
	Uninjected	AAV+Veh	AAV+D-DPTIP	Uninjected	AAV+Veh	AAV+D-DPTIP
ALP (U/L)	67.8±4.66	72.0±6.08	78.8±13.55	109.3±13.45	119±17.12	96±25.21
AST (U/L)	50.4±15.09	65±15.12	54.4±14.31	70.75±13.72	87±25.89	68.4±25.14
ALT (U/L)	30.6±4.28	26.4±5.25	30.4±6.88	26±4.76	23.2±8.01	19.4±4.83
Creatine kinase (U/L)	114±86.12	186±75.70	97.2±42.57	178.3±64.06	222±130.20	182±127.60
Albumin (g/dL)	2.72±0.11	2.96±0.05	2.86±0.15	3.075±0.05	3.04±0.11	2.92±0.13
Total Bilirubin (mg/dL)	0.10	0.10	0.10	0.175±0.05	0.10	0.12±0.04
Total Protein (g/dL)	4.7±0.14	4.92±0.13	5±0.12	4.8±0.08	4.8±0.16	4.82±0.16
Globulin (g/dL)	1.98±0.04	1.96±0.09	2.14±0.05	1.73±0.05	1.76±0.11	1.9±0.07
Bilirubin - Conjugated (mg/dL)	0	0.02±0.045	0	0	0	0
BUN (mg/dL)	21.6±1.82	21.4±2.07	30.8±4.32	17.75±1.89	17.8±1.48	25.2±8.87
Creatinine (mg/dL)	0.10	0.06±0.05	0.1±0.07	0.025±0.05	0.08±0.04	0.04±0.05
Cholesterol (mg/dL)	83±4.69	111±7.07	108.8±10.01	71.25±1.71	84.4±4.72	92±7.91
Glucose (mg/dL)	269.4±22.88	234.2±63.19	238.8±34.95	252.3±34.51	234.8±34.36	224±49.39
Calcium (mg/dL)	9.54±0.11	9.5±0.26	9.58±0.47	9.43±0.22	9.56±0.15	9.48±0.22
Phosphorus (mg/dL)	7.06±0.93	6.76±0.31	7.22±1.11	8.15±0.98	7.5±1.63	7.02±1.35
Bicarbonate TCO2 (mmol/L)	22.6±2.88	23±2.45	25.4±3.13	20.75±2.22	24±2.55	21.2±2.28
Chloride (mmol/L)	106.6±0.89	109.8±0.45	109.3±1.71	110±0.82	110.2±1.48	112.3±0.58
Potassium (mmol/L)	5.36±0.52	5.24±0.47	5.025±0.41	5.425±0.05	5.08±0.46	5.3±0.44
ALB/GLOB ratio	1.38±0.04	1.5±0.07	1.34±0.09	1.775±0.05	1.74±0.15	1.56±0.09
Sodium (mmol/L)	153.6±0.55	155.8±0.45	155.8±0.96	153.3±0.96	154±0.71	152.3±0.58
Bilirubin - Unconjugated (mg/dL)	0.10	0.08±0.045	0.10	0.18±0.05	0.10	0.12±0.045
NA/K Ratio	28.8±2.86	29.8±2.78	31±2.45	28.25±0.50	30.4±2.61	29±2.65

Figure S10. D-DPTIP is well tolerated in AAV-hTau mice. **A**-**B**) Weekly body weight changes from baseline from male (**A**) and female (**B**) mice. Points represent mean ± SEM. **C**) Clinical chemistry parameters from uninjected C57BL6/J control mice, AAV-hTau vehicle treated mice, and AAV-hTau D-DPTIP treated mice from both males and females. Values represent mean ± SD.
